# The effects of EFL wordlist and proficiency on vocabulary knowledge

**DOI:** 10.3389/fpsyg.2024.1289106

**Published:** 2024-06-20

**Authors:** Zhen Bao, Cheng Peng

**Affiliations:** School of Foreign Languages, Shanghai Jiao Tong University, Shanghai, China

**Keywords:** the Gaokao Word List, L2 proficiency, vocabulary knowledge, wordlist effect, word recognition

## Abstract

**Introduction:**

The Gaokao Word List (GWL) in China serves as a guideline for learning L2 vocabulary, but there are few studies verifying its effect on university EFL learners’ vocabulary knowledge.

**Method:**

This study investigated the effects of the GWL and EFL proficiency on 66 Chinese university EFL learners’ vocabulary knowledge by administering word recognition tests.

**Results and discussion:**

The results showed that: (1) the GWL had significant effects on participants’ receptive vocabulary knowledge; (2) EFL proficiency had significant effects on participants’ word recognition, without interaction with the GWL. These findings were discussed through the lens of frequency of exposure, accounting for the overwhelming GWL effect on learners’ vocabulary knowledge. We suggest EFL proficiency be taken into consideration when the GWL is revised in the future, to smoothen the transition in vocabulary learning from high school to university, and improve vocabulary learning efficiency.

## 1 Introduction

Vocabulary is crucial to foreign language teaching and learning. As suggested by Meara (1996), learners with larger vocabulary are more proficient in various language skills than those with smaller vocabulary. However, words in a language are too numerous to acquire, especially for L2 learners. Considering the workings of human memory and the tendency to forget information over time, long-term retention of newly-learnt vocabulary poses great challenges for learners. The majority of forgetting occurs shortly after initial learning, with a decrease in the rate of forgetting thereafter (Schmitt, 2010b). It is recommended to increase the frequency of exposure so as to retain more words in memory. To repeatedly encounter target words, West et al. (1934) introduced the concept of “vocabulary selection,” and wordlist is the realized product of such concept. In high schools in China, the Gaokao Word List (GWL) is deemed as the framework for foreign language teaching and learning, since the Gaokao, or the National College Entrance Examination in China, is a standardized test taken by over 10 million students in their final year of high school each year to gain admission to universities. Due to the competitiveness of the life-changing exam, students undergo extensive preparation to excel in this high-stakes examination, reflecting the importance of this exam in shaping students’ future prospects. In practice, teachers usually follow the GWL in their routine vocabulary instruction to expand students’ vocabulary in preparation for the Gaokao (Gu and Li, 2009; Bao and Xu, 2022).

Although the GWL plays a guiding role in vocabulary learning, the extent of its influence is unknown. It remains to be investigated whether the vocabulary knowledge of university EFL learners, who have graduated from high schools for over 3 years, still displays the influence of the GWL. In EFL learning contexts, is it likely that advanced EFL learners are immune to the effect of the GWL, whereas lower-proficiency learners are more prone to the GWL effect? In response, this study examined the effects of the GWL and L2 proficiency on EFL learners’ vocabulary knowledge, so as to see whether the GWL effect on learners’ vocabulary knowledge is long-lasting, and whether learners of different proficiency levels react differently to the GWL.

## 2 Literature review

### 2.1 Word list

In EFL learning, vocabulary is considered to be of utmost importance. Nevertheless, a significant portion of EFL students encounter difficulties due to inadequate exposure to target language vocabulary, whereas repeated exposure to the target vocabulary is deemed essential for the effective acquisition of new words (Webb, 2007). Previous studies indicate that learners may need between 5 to 16 or more exposures to successfully learn a new word (Nation, 1990). Although some new words may be acquired incidentally, the vocabulary knowledge is often not deeply entrenched (Pigada and Schmitt, 2006). In contrast, intentional vocabulary learning involves explicit instruction and repeated focus on words by learners. It has been shown when learners consciously concentrate on acquiring vocabulary, the retention rate is higher compared to incidental learning (Schmitt, 2010b).

Many EFL learners equate language learning with intentional vocabulary learning, investing considerable time in memorizing words from wordlists (Read, 2010), in an attempt to increase exposure to target words. Wordlists, commonly used tools for explicit vocabulary learning, are typically created based on specific criteria to facilitate English learners’ vocabulary development (Nation, 2016). When it comes to developing vocabulary knowledge, the utilization of wordlists is highly beneficial to EFL learners with limited exposure to the target language (Read, 2010; Yamamoto, 2014). Depending on their design, wordlists can serve at least two heuristic functions. Firstly, a wordlist can act as a reflection of what learners have learned, acting as a representation of their knowledge. Secondly, a wordlist can function as a guide for what learners should learn (Pinchbeck et al., 2022).

English, being one of the mandatory subjects in high school, holds a pivotal role in the Gaokao. The Ministry of Education of China (2017) has established definitive guidelines for English learning and evaluation, i.e., the English Curriculum Standards for High Schools, which outlines the vocabulary to be taught, learned, and evaluated in the Gaokao. In accordance with the official Gaokao Word List, high school graduates should aim to acquire around 3,000 basic words. The GWL, compiled by language educators with expertise in EFL teaching, serves as a guide for students by focusing on common expressions related to various aspects of daily life (Bao and Xu, 2022). Despite indications that the selected words are relevant to commonly discussed topics, the specific criteria for word selection were not clearly reported in the introduction of the GWL. By calculating the coverage of the GWL words in the BNC/COCA frequency-based wordlists (i.e., a series of 1,000-word lists based on frequency level) (Nation, 2016), we found that 79.37% of the GWL words are covered in the first 3,000 words (i.e., 41.69% in the 1^st^ 1,000 words, 27.92% in the 2^nd^ 2000 words, 9.76% in the 3^rd^ 1,000 words), indicating that frequency could be the basic criterion for selecting words on the GWL.

The GWL was created to assist students in preparing for the Gaokao English exam, serving as a crucial reference for curriculum design, word selection in learning materials, classroom instruction, and language assessments (Sun, 2005; Gu and Li, 2009; Bao and Xu, 2022). Language-focused education in China typically involves explicit vocabulary instruction, with the official wordlist being a valuable resource for such endeavors (Sun, 2005). High school students in China commonly employ the wordlist learning strategy (Qian, 2020), spending a large amount of time memorizing words from the GWL in preparation for the Gaokao exam. Teachers often provide explicit vocabulary instruction and frequent quizzes based on the wordlist to monitor students’ progress in vocabulary knowledge (Zhang, 2015). Additionally, the construction of the Gaokao exam heavily relies on the GWL, which serves as a primary guideline for English testing. Nation (2016) stressed the importance of empirical studies on influential wordlists. Reynolds et al. (2018) discovered that wordlists designed for high school students have a lasting impact on university students’ vocabulary knowledge, with listed words significantly outperforming off-list words. Despite the widely recognized influence of the GWL on vocabulary learning and teaching, there is a lack of empirical evidence verifying its effects.

### 2.2 Vocabulary knowledge assessment

Vocabulary knowledge can be categorized into receptive (passive) and productive (active) dimensions (Henriksen, 1999; Laufer and Goldstein, 2004; Milton, 2009; Schmitt, 2010a). Receptive vocabulary knowledge involves recognizing the form of a word (Laufer et al., 2004), or providing its synonym or translation in one’s first language (Webb, 2009), while productive vocabulary knowledge is often defined as the ability to recall both the form and meaning of words (Laufer et al., 2004; Webb, 2008). Nation (2013) expanded this view by considering vocabulary knowledge as multifaceted, encompassing form (e.g., pronunciation, spelling), meaning (e.g., form/meaning relationships, associations), and use (e.g., grammatical functions, collocations). However, in vocabulary research, the focus tends to be on the form and meaning of words (Laufer and Goldstein, 2004; Zhong, 2018), with many studies operationalizing vocabulary breadth knowledge as knowledge of form-meaning mapping (Laufer and Goldstein, 2004; Schmitt, 2014).

The testing of form-meaning mapping knowledge falls into four categories: passive recognition test, passive recall test, active recall test, and active recognition test (Laufer et al., 2004; Laufer and Goldstein, 2004; Schmitt, 2010a). Passive recognition test is a commonly utilized format that assesses learners’ vocabulary knowledge by requiring learners to demonstrate that they know the target language word by recognizing the form of a word or selecting the corresponding L1 translation from provided options. This test format is considered fundamental in evaluating word knowledge (Milton, 2009). On the other hand, passive recall test requires learners to supply the L1 translation of an L2 word (Laufer and Goldstein, 2004; Schmitt, 2010a). In contrast to passive tests, active recall test prompts learners to provide the L2 target word in response to its L1 translation. Another vocabulary test format is the active recognition test, where learners must choose the corresponding L2 word when given the L1 translation. These assessment formats typically involve translation equivalents (Laufer and Goldstein, 2004). However, the use of translation as a teaching or testing tool is not always liked. Communicative approaches to language teaching favor gap-fill tests where learners are instructed to fill in missing words in sentences to demonstrate their knowledge of the target words (Milton, 2009).

The assessment of vocabulary knowledge was moderated by test format (Bowles and Salthouse, 2008). For example, tasks requiring the recall of lexical meanings and forms, such as word definition tasks, show greater susceptibility to individual differences compared to word recognition tasks, which only involves recognizing the forms of the L2 words (Verhaeghen, 2003; Bowles and Salthouse, 2008). Word recognition tasks do not require retrieving the target word from memory, thereby helping to mitigate the confounding effect of individual differences. In this study, the vocabulary knowledge of EFL learners will be assessed using word recognition task.

The Yes/No vocabulary test is a passive recognition test format that has been extensively researched (Milton, 2009). This test format is designed to gauge learners’ receptive vocabulary through a simple judgment of whether a given lexical item is known or not (Meara, 1996). Within the vocabulary testing literature, Yes/No vocabulary tests have been proposed as easy alternatives to multiple-choice tests (Meara and Buxton, 1987). It has been utilized in the large-scale European Dialang project for assessing vocabulary proficiency in 14 European languages.^1^ Compared to other assessment methods, the Yes/No format offers the advantage of being quick and easy to construct the vocabulary test. Since participants are less likely to feel bored and lose concentration in a short time span, the test results are likely to be more reliable (Milton, 2009).

There are also criticisms regarding the limitations of the vocabulary test format. One issue is that learners’ self-report of their knowledge of a word may not be an accurate reflection of their actual vocabulary knowledge (Nation, 1990). When learners do not really recognize a word or are not sure, they might guess. Lucky guesses may lead to an overestimation of the learners’ true vocabulary knowledge (Urdaniz and Skoufaki, 2022). To address the potential guesswork, pseudowords are often included in vocabulary tests. Pseudowords are fabricated words that resemble real words but are not actually part of the language (Milton, 2009). In this study, we utilized pseudowords generated by Wuggy (Keuleers and Brysbaert, 2010) to take into consideration the possibility that certain learners might overestimate their knowledge. Another issue is that the Yes/No test format may pose challenges for individuals with dyslexia or those whose L1 and L2 are cognate languages (Beeckmans et al., 2001). Cognates have typically been described in same-script language pairs as words that share similar form and meaning (Dijkstra, 2007). Studies involving different-script languages have also used the term “cognate” to indicate phonologically similar lexical pairs (Kim and Davis, 2003; Hoshino and Kroll, 2008). There exist a few Chinese-English cognates, which are transliterated loan words, with their pronunciations as similar as possible to their English counterparts (Wen and van Heuven, 2017). To address these concerns, we excluded transliterated loanwords from our experimental stimuli and recruited Mandarin-speaking university students without dyslexia to participate in the study.

Several studies have confirmed the efficacy of the Yes/No test format. Mochida and Harrington (2006) utilized the same lexical items in both the Yes/No test and the Vocabulary Levels Test (VLT) to allow a direct comparison of test performance. They found that performance on the Yes/No test could reliably predict scores on the VLT among EFL university students. The various scoring methods for the Yes/No test all demonstrated strong predictive power for VLT performance, with correlation coefficients exceeding 0.85. Regardless of the scoring method used, the Yes/No test performance accounted for more than 75% of the variance in VLT scores. Also, Meara (1996) observed a moderately strong correlation, around 0.7, between Yes/No test performance and traditional multiple-choice tests taken by EFL learners. Further supporting the efficacy of the Yes/No test, Lemhöfer and Broersma (2012) developed the LexTALE, a validated Yes/No vocabulary measure that reliably assesses English vocabulary knowledge among medium- to high-proficient EFL learners. The relevant literature findings confirm the reliability and validity of the Yes/No test as a measure of L2 receptive vocabulary knowledge, prompting the current study to adopt the Yes/No vocabulary test format to evaluate learners’ receptive vocabulary knowledge.

### 2.3 L2 proficiency and vocabulary knowledge

The importance of English proficiency in vocabulary acquisition is well-established in relevant literature, with vocabulary knowledge often seen as a key indicator of overall L2 proficiency (Alderson, 2005; Mitsugi, 2018; Zeng et al., 2019). Variations in L2 proficiency can significantly influence learners’ vocabulary retention and word recall abilities (Agustin-Llach, 2022). Previous research has shown that learners’ proficiency levels play a crucial role in their vocabulary learning outcomes. For example, Teng (2022) identified English proficiency as a notable predictor of vocabulary learning performance, aligning with findings from Tekmen and Daloǧlu’s (2006) study, wherein advanced learners acquired a significantly higher number of words compared to those with lower proficiency levels. Moreover, studies have consistently demonstrated a positive relationship between L2 proficiency and vocabulary knowledge. Studies by Masrai (2023) and Zareva et al. (2005) have highlighted a strong correlation between vocabulary knowledge and overall language proficiency. Specifically, Milton et al. (2010) revealed significant positive associations between written and aural vocabulary knowledge and the performance of L2 learners in various language skills such as writing, reading, listening, and speaking.

During the initial stages of language acquisition, L2 learners are prone to be negatively affected by their first language, which can hinder the acquisition of L2 vocabulary. The similarities in structure between learners’ L1 and L2 can have an impact on the ease with which vocabulary is learned (Crystal, 1987). It is suggested that learning a second language that shares structural similarities with one’s L1 may be easier compared to learning a language that is structurally different. For example, the comprehension and production of words containing phonemes that are absent in the learner’s L1 can present difficulties (Laufer, 1990). However, as L2 proficiency improves, learners will concurrently incorporate more sophisticated lexical items in their language use. A longitudinal study has confirmed the influence of proficiency levels on vocabulary acquisition (Salsbury et al., 2011).

While extensive research exists on the relationship between vocabulary knowledge and L2 proficiency, limited attention has been given to investigating how proficiency levels influence vocabulary acquisition when learners are guided by an official wordlist. It remains largely unknown whether learners with varying proficiency levels respond similarly to the guided wordlist (i.e., the GWL). Therefore, it is imperative to examine how L2 proficiency influences vocabulary acquisition when guided by the GWL.

### 2.4 The current study

The GWL was compiled to assist vocabulary learning and teaching during high school. We hypothesized that the GWL exerts significant effects on EFL learners’ vocabulary knowledge, even long after they enter universities. Whether university EFL learners’ vocabulary knowledge is still shaped or constrained by the high-school GWL after over 3 years in university remains to be revealed. However, few studies have been conducted to confirm the washback effect of the GWL on the vocabulary knowledge of university students, and to provide empirical evidence for the revision or compilation of the wordlist. In classroom settings, the GWL presents all target words to learners of different proficiency levels. During vocabulary learning process, whether learners’ proficiency levels interact with the GWL influence is still unknown to us. We thus have no idea whether compiling graded wordlists tailored to learners of different proficiency levels is advisable. Nevertheless, few studies have investigated whether L2 proficiency affects wordlist-based vocabulary learning. Thus, by consulting Brysbaert et al.’s (2021) standard online lexical recognition paradigm featuring good reliability and validity in evaluating learners’ vocabulary knowledge, this study explores the effects of an official wordlist for high school learners and L2 proficiency on vocabulary knowledge of university EFL learners across China.

The research questions are as follows:

1. Does the GWL have long-lasting effects on EFL learners’ vocabulary knowledge?
2. Does L2 proficiency have any impact on EFL learners’ wordlist-based vocabulary learning?

## 3 Method

### 3.1 Participants

A total of 66 university students with Mandarin Chinese as their L1, who had taken the Gaokao, were recruited as participants via online crowdsourcing in the present study. The sampling method has been frequently adopted in previous seminal studies to access participants from diverse geographical areas, thus enhancing the sample’s representativeness (e.g., Kuperman et al., 2012; Warriner et al., 2013; Brysbaert et al., 2014). The participants come from various regions in China, including East China, Central China, South China, North China, Northeast, Northwest, and Southwest. Participants completed a set of vocabulary recognition tests, which contained 30 pseudowords to help detect participants’ noncompliance with task instructions or inattentiveness to the task. Following the practice by Brysbaert et al. (2021) to encourage faithful responses, we explicitly warned the participants that both true words and pseudowords were included and that “*Yes*” responses to pseudowords would result in penalty points. To ensure data quality, an automatic filter was implemented so that surveys with more than five “*Yes*” responses to pseudowords were excluded from the final dataset, which is a common practice in word recognition task. As a result, 24 participants were removed. Participants who gave “N” response (i.e., “I do not know the meaning of this word”) to all real and pseudo words were also excluded from analysis. Accordingly, two participants were filtered out. The final sample included 40 participants (62.5% female), with their mean age being 20.68 years (SD = 1.68). The majority of participants in the study are either junior (27.5%) or senior (50%) university students, with sophomores and freshmen making up 17.5 and 5% of the sample, respectively.

### 3.2 Experimental materials

The vocabulary recognition tests employed in this study contained 35 GWL listed words, 35 matched words outside the GWL (referred to as “the matched off-list words”), and 30 pseudowords (Note. the rationale for using pseudowords in word recognition test has been clarified in section *2.2 Vocabulary Knowledge Assessment*). Specifically, the procedure of word set construction was as follows.

Firstly, we imported the following information for the 2,070 words on the GWL: word frequency, dominant part of speech, and word concreteness. As the GWL encompasses general English words used in everyday interactions (Bao and Xu, 2022), we opted to retrieve word frequency from the SUBTLEX-US corpus, which is derived from subtitles of movies and TV shows and contains 51 million word tokens from 8,388 subtitle files (Brysbaert and New, 2009). Traditionally, corpora were primarily sourced from books, newspapers, and magazines, which often cover themes not commonly discussed in daily life (Brysbaert and New, 2009). Conversely, subtitles from films and television offer a more realistic representation of everyday language usage (New et al., 2007). In addition, research on American English and other languages has indicated that word frequencies based on film and television subtitles are better predictors of word processing efficiency than word frequencies based on books and other written sources (e.g., Brysbaert and New, 2009; Cai and Brysbaert, 2010; Keuleers et al., 2010).

We utilized the Zipf value of word frequency, which is measured on a logarithmic scale ranging from 1 (representing low frequency) to around 7 (representing high frequency). The Zipf value is calculated using the formula log10 (frequency per billion words), where a Zipf value of 1 corresponds to a word frequency of 10 per billion words and a Zipf value of 2 corresponds to a word frequency of 100 per billion words, and so on (van Heuven et al., 2014). The Zipf value was chosen in lieu of using raw frequency data for two reasons. Firstly, the size of a corpus can skew raw frequency data. Secondly, the raw frequency effect tends to follow a logarithmic curve, thereby amplifying the differences between words with similar frequencies (Brysbaert et al., 2018).

Secondly, the dominant part-of-speech (PoS) information was sourced from the SUBTLEX-US corpus. Within the SUBTLEX-US corpus, the CLAWS algorithm, developed at Lancaster University, was employed to assign PoS tags to words. The accuracy of the CLAWS tagger has been validated through its application to other corpora (e.g., the British National Corpus and the Corpus of Contemporary American English), achieving accuracy rates between 96 and 97% (Brysbaert et al., 2012). The SUBTLEX-derived PoS tags come with information on how frequent each word form is used as a particular part of speech (Strik Lievers et al., 2021). Furthermore, the PoS tags obtained from this corpus comprise one of the most comprehensive sets of part-of-speech tags readily available for statistical analysis (Brysbaert et al., 2012).

Thirdly, word concreteness values were extracted from the largest database of word concreteness ratings available (Brysbaert et al., 2014). A total of over 4,000 fluent English-speaking adults were invited to rate 37,058 words on a scale ranging from 1 (representing high abstractness) to 5 (representing high concreteness). The correlation between the concreteness ratings provided by the MRC database (Coltheart, 1981) and those by Brysbaert et al. (2014) was notably strong (*r* = 0.919), highlighting the validity of the concreteness ratings.

Lastly, 219 words that do not appear in the GWL were sampled matching the GWL words on part-of-speech, word length [*t* (68) = 0.000, *p* = 1], word frequency [*t* (68) = 0.127, *p* = 0.900], number of morphemes [*t* (68) = 0.000, *p* = 1], orthographic similarity [*t* (68) = 0.127, *p* = 0.889], and concreteness [*t* (68) = 0.051, *p* = 0.960]. A series of independent samples t-tests have shown that there are no significant differences between the two sets of words in terms of the lexico-semantic variables, as shown in the above square brackets. The descriptive data for the two word subsets are displayed in Table 1. Then, we randomly selected 35 word pairs from the 219 pairs of word stimulus.

**Table 1:** Descriptive data for the two word subsets.

Word subset	Lexical characteristics	Mean	SD.	Max.	Min.
The GWL listed words (*n* = 35)	Frequency	3.82	0.60	4.64	2.07
	Concreteness	3.13	1.07	5.00	1.52
	Word length	6.77	2.60	13	3
	Number of morphemes	1.57	0.70	3	1
	Orthographic similarity	2.42	0.90	4.95	1.00
The matched off-list words (*n* = 35)	Frequency	3.80	0.65	5.41	2.13
	Concreteness	3.12	1.09	5.00	1.54
	Word length	6.77	2.60	13	3
	Number of morphemes	1.57	0.66	3	1
	Orthographic similarity	2.45	1.15	6.20	1.00

Orthographic similarity is measured by the orthographic Levenshtein distance 20 (Yarkoni et al., 2008).

Our word recognition task required the participants to indicate whether they knew each item by selecting “*Yes*” or “*No*.” This is a standard online word recognition paradigm, which has shown good reliability and validity in assessing participants’ vocabulary knowledge (Brysbaert et al., 2021). The inter-rater reliability as measured by Intraclass Correlation Coefficient for the vocabulary recognition tests was 0.869, indicating the vocabulary tests are reliable. In addition, to check the consistency of results across different participants, we tested another group of university EFL learners using the same word recognition task (N = 44, average age = 20.43 years). The Pearson correlation between the two groups’ vocabulary recognition performance was significantly strong (*r* = 0.892, *p* < 0.001), which suggests the reliability of the vocabulary test.

The English proficiency test used in this study was adapted from the Syndicate (2001). Our pilot study suggested that selecting 10 multiple-choice items and 10 cloze items (i.e., the total score is 20) was most suitable for an online survey paradigm, balancing test time and effectiveness. The internal reliability of this proficiency test, as calculated by Cronbach’s Alpha, was 0.779, indicating a high level of reliability.

### 3.3 Experimental procedures

The online testing was comprised of vocabulary recognition tests and English proficiency test. Participants were assigned to complete the vocabulary recognition test by selecting “*Yes*” or “*No*.” Subsequently, participants completed the English proficiency test and provided demographic information.

### 3.4 Statistical analysis

To answer the two research questions, we conducted linear mixed-effects (LME) modeling with the lme4 package in R because it allows including participant as random effect, which enables us to account for individual differences. We included the following variables as fixed effects in the LME model: L2 proficiency, word source (listed words vs. off-list words), and the interaction between proficiency and word source. The recognition rate was the dependent variable. Finally, effect sizes were calculated for the model using the MUMIn function. MUMIn provides *R^2^* values for the fitted model in two forms. Marginal *R^2^* values are associated with the fixed effects, while conditional *R^2^* values reflect both the fixed and the random effects combined.

## 4 Results (the effects of the GWL and L2 proficiency on vocabulary knowledge)

Descriptive statistics showed that the average L2 proficiency of the 40 participants was 12.175 (SD = 4.408). The recognition rates for these two sets of words are shown in Table 2. The vocabulary recognition rates of the GWL listed words were higher than those of the off-list words.

**Table 2:** Recognition rates.

	Mean	SD.	Lower CL	Upper CL
The GWL listed words	0.833	0.150	0.784	0.882
The matched off-list words	0.478	0.176	0.429	0.527

SD, standard deviation, CL, confidence limit.

The results of the linear mixed-effects model are presented in Table 3. Considering the *R^2^* values (*R^2^* marginal = 0.594, *R^2^* conditional = 0.907), this model seems to explain a substantial amount of variance in learners’ word recognition performance. The LME results indicate that both word source and L2 proficiency have significant effects on participants’ receptive knowledge of general English words.

**Table 3:** The LME model for recognition rates.

	Estimate	SE	df	*t*-value	Pr(>|*t*|)
(Intercept)	0.676	0.073	47.620	9.271	< 0.01^**^
Proficiency	0.013	0.006	47.620	2.287	0.027^*^
The matched off-list words	−0.363	0.049	38	−7.369	< 0.01^**^
Proficiency: The matched off-list words	0.001	0.004	38	0.168	0.867

^**^p < 0.01, ^*^p < 0.05. The matched off-list vocabulary was the reference level of the word source. R^2^ marginal = 0.594. *R*^2^ conditional = 0.907.

As for the first research question, the results reveal that a significant difference in word recognition is observed between the GWL listed words and the matched off-list words, (*F* (1, 38) = 54.306, Pr(>F) < 0.001). The result indicates that the recognition rates of the words in the GWL are significantly higher than those of the words outside the list. Our result is consistent with the findings by Reynolds et al. (2018) that the reference wordlist in high school has a significant impact on university learners’ vocabulary knowledge.

As for the second research question, further analysis using the anova() function reveals that L2 proficiency makes a positive and significant contribution to participants’ vocabulary knowledge of general English words, (*F* (1, 38) = 6.198, Pr(>F) = 0.017). Our results indicate that vocabulary knowledge tends to develop concurrently with the improvement of L2 proficiency. Learners of higher proficiency boast a larger vocabulary than do learners of lower proficiency. The LME results reveal that there is no interaction effect of L2 proficiency by word source on word recognition, (*F* (1, 38) = 0.028, Pr(>F) = 0.867).

## 5 Discussion

By manipulating the word source (on-list words vs. off-list words), our study revealed that both GWL and L2 proficiency have significant effects on learners’ vocabulary knowledge, without interaction. After controlling for other lexical variables, the GWL listed words were recognized more accurately than their off-list counterparts. Even after learners have studied in universities for over 3 years, the GWL effect on learners’ vocabulary knowledge is still robust. Under the guidance of the GWL, L2 proficiency still significantly influenced learners’ vocabulary knowledge. Learners of higher proficiency boast more vocabulary knowledge than do learners of lower proficiency. We discussed our study results on the basis of relevant psycholinguistic theories.

### 5.1 The wordlist effect on vocabulary knowledge

The GWL has a significant effect on vocabulary recognition, consistent with the findings by Reynolds et al. (2018) that processing words within wordlist is superior to that outside the list. The participants in this study were university students, most of whom were juniors or seniors. Even after 3 years or more, their knowledge of the GWL listed words learned in high school remained significantly superior to that of the off-list words. This finding suggests that not only does the wordlist influence EFL vocabulary learning in high school, but it also has a strong and long-lasting effect on university learners, in line with the findings of Reynolds et al. (2018), which examined the washback effect of an official reference wordlist compiled for high school learners to prepare for the College Entrance Examination. They revealed the significant influence of the wordlist on the vocabulary learning and retention of EFL university students, indicating the long-lasting influence of official reference wordlists.

The wordlist authorized by the national education authority plays a significant role in language education, including teaching, learning, assessment, and curriculum development (Sun, 2005; Bao and Xu, 2022). In practice, textbooks, classroom instruction, and language tests aim to incorporate as many words from the official list as possible (Nation, 2016). English teaching and learning materials developed based on the wordlist are the primary source of input for high school students in China (Gu and Li, 2009), where achieving a high score on the Gaokao exam is crucial for admission to top universities. Teaching and learning strategies in Chinese high schools are influenced by the wordlist and test-taking objectives, with a focus on preparing students for the Gaokao exam (Bao and Xu, 2022). Students dedicate most of their English learning time to materials that align with the Gaokao exam (Sun, 2005; Gu and Li, 2009). Through repeated exposure to the listed words in various contexts, learners are more likely to acquire and retain these listed words. Research has shown that encountering a word at least eight times increases the likelihood of acquisition (Gullberg et al., 2012; Elgort et al., 2018; Peters and Webb, 2018), highlighting the importance of repeated exposure in vocabulary learning (Nation, 2014). Thus, the official wordlist brings about learners’ repeated encounters with the listed words, which facilitates the acquisition of these words, as evidenced by significantly higher recognition rates of the listed words compared to words not included in the list.

The interpretation of the effect that frequency of exposure has on vocabulary acquisition is straightforward: the more often a learner is exposed to any learning material, the stronger the memory and the more likely the material will be learned and retained over time (Ellis, 2012). According to the lexical entrenchment hypothesis (Diependaele et al., 2013), the process of acquiring words is driven by exposure. The total number of exposures to words, which accumulates over time, reflects a person’s experience and usage of those words. Variation in exposure to words in a language is the main factor that determines word processing, both in L1 and L2 (Brysbaert et al., 2017). The recognition differences between the GWL listed words and the off-list words can be explained by variations in exposure to these two sets of words. Through repeated encounters with the listed words, the quality of their vocabulary knowledge is enhanced (Nation, 2013), and the links between the form and meaning representations of these listed words are stronger than those of the off-list words. After learners repeatedly encountered the GWL listed words, the representations of these listed words were entrenched in mental lexicon and were retained over time. With the mapping between lexical form and meaning reinforced, learners can accurately activate the corresponding conceptual representations in recognition tests. By contrast, the off-list words are exposed far less, which leads to less efficient word recognition. Under the guidance of the GWL, students engage in learning the same set of words from textbooks, consult the GWL or dictionaries, encounter them in their readings, and repeatedly practice them. While this intensive approach may foster a deep comprehension of the listed vocabulary, excessive focus on a limited number of words could potentially limit learners’ motivation or ability to acquire a broader range of vocabulary (Schmitt, 2014).

### 5.2 The L2 proficiency effect on wordlist-based vocabulary knowledge

The L2 proficiency has a significant impact on participants’ ability to recognize vocabulary, which supports previous research findings (e.g., Zeng et al., 2019; Agustin-Llach, 2022; Teng, 2022) that English proficiency serves as a strong predictor of vocabulary knowledge or word recognition performance.

On the one hand, as learners’ English proficiency improves and vocabulary exposure accumulates, their semantic network becomes richer, resulting in improved vocabulary knowledge (Schmitt, 2014; Enayat and Derakhshan, 2021). Learners with higher proficiency are able to respond accurately when words are presented, whereas those with lower proficiency may provide incorrect responses due to erroneous connections between lemma (i.e., semantic and syntactic information about words) and lexeme (i.e., morphological and formal information about words), or weak connections that fail to activate the corresponding lemma information (Jiang, 2000). On the other hand, morphological awareness (MA) varies among learners of different proficiency levels. Higher-proficiency learners possess strong MA, which allows them to process target words efficiently, particularly words with complex morphology (Zeng et al., 2019). Nagy et al. (2014) suggest that learners with strong MA are able to decode morphologically complex words, such as derived words and compound words, by utilizing both morphological and semantic channels. In contrast, learners with weak MA may have difficulty recognizing morphologically complex words due to blockage or absence of these channels.

There is no interaction between L2 proficiency and the wordlist, indicating that the wordlist has beneficial impacts on vocabulary knowledge of learners with varying proficiency levels, consistent with findings by Yamamoto (2014). Lower-proficiency learners are significantly poorer at recognizing words than their higher-proficiency counterparts, even for the GWL listed words. Vocabulary knowledge contributes to improving overall language proficiency, which in turn affects learners’ vocabulary recognition outcomes (Laufer and Nation, 1995; Nation, 2013). Constrained by language proficiency, lower-proficiency learners tend to acquire poor or incomplete vocabulary knowledge (e.g., inaccurate lexeme information, lack of lemma information, lack of links between lemma and lexeme), even when guided by the same wordlist. With higher demands for future academic development, learners with higher proficiency should expand their vocabulary knowledge beyond the current scope of the GWL. Their repertoire of words should include more advanced vocabulary appropriate for their level of proficiency and future academic growth. A single reference wordlist for learners with different proficiency levels might disregard the variations in their demands of language development, and might impede their ability to effectively expand their vocabulary knowledge. To address the issue, Nation (2016) proposed that wordlist compilers should create graded wordlists that align with learners’ language proficiency levels. Thus, we propose that future revisors of the GWL should take into account the L2 proficiency of high school students across the country and develop graded wordlists that cater to learners at different proficiency levels.

Last but not least, our results (i.e., both wordlist and L2 proficiency significantly influence EFL learners’ vocabulary knowledge), together with those of Reynolds et al. (2018) can be generalized to EFL wordlists outside China. That is, in EFL learning contexts where target language input is limited, when assisted or guided by a wordlist, EFL learners’ vocabulary knowledge will be greatly influenced or shaped by the wordlist. With learners’ repeated exposure to the listed vocabulary, the knowledge of the listed words becomes more entrenched in learners’ mental lexicon than the off-list words over a long period of time. When developing wordlists for EFL learners, wordlist compilers or revisors should take wordlist’ long-term washback effects and L2 proficiency effect into consideration.

## 6 Conclusion

By manipulating word source (i.e., included in the GWL or not), we conducted an online vocabulary knowledge test to examine the effects of the GWL and L2 proficiency on university EFL learners’ word recognition performance. Our study revealed that the GWL had a significant and long-lasting effect on the vocabulary knowledge of university EFL learners in China, with the recognition rates of the list words being significantly higher than those of the off-list words. Moreover, L2 proficiency had a significant effect on vocabulary recognition, and had no interaction with the GWL. Be the words on-list or off-list, the word recognition performance of higher-proficiency participants was significantly better than that of lower-proficiency participants.

Our findings verify the significant guiding role of wordlists in EFL vocabulary learning. In EFL learning environments with limited target language input, wordlists specify the target vocabulary for learners, making vocabulary teaching/learning more efficient and straightforward. In addition, the long-term washback effect of wordlist has been confirmed, with knowledge of the listed words entrenched in learners’ mental lexicon. Even after 3 years or more, learners’ vocabulary knowledge within the scope of wordlist is still significantly superior to that outside the list. To improve EFL learners’ vocabulary learning efficiency, wordlist compilers should consider users’ L2 proficiency so that graded wordlists tailored to various proficiency levels can be developed.

There are some limitations in this study. Since we adopted the online recognition paradigm to reach participants from various regions in China, we did not collect the reaction time data, which might also reflect the effects of the variables concerned on vocabulary recognition performance.

## Data availability statement

The original contributions presented in the study are included in the article/supplementary material, further inquiries can be directed to the corresponding author.

## Ethics statement

Ethical approval was not required for the studies involving humans in accordance with the local legislation and institutional requirements. The studies were conducted in accordance with the local legislation and institutional requirements. The participants provided their written informed consent to participate in this study.

## Author contributions

ZB: Conceptualization, Data curation, Formal analysis, Investigation, Methodology, Validation, Visualization, Writing – original draft, Writing – review & editing. CP: Conceptualization, Formal analysis, Funding acquisition, Project administration, Resources, Software, Supervision, Writing – review & editing.

## Footnotes

^1^http://www.dialang.org
